# Synthesis of Silicon Nitride Nanoparticles by Upcycling Silicon Wafer Waste Using Thermal Plasma Jets

**DOI:** 10.3390/ma15248796

**Published:** 2022-12-09

**Authors:** Tae-Hee Kim, Seungjun Lee, Dong-Wha Park

**Affiliations:** 1 Department of Chemical Engineering, Wonkwang University, Iksan 54538, Republic of Korea; 2 Department of Chemistry and Chemical Engineering, Inha University, Incheon 22212, Republic of Korea

**Keywords:** silicon waste, silicon nitride, recycling, upcycling, thermal plasma, nanoparticle synthesis

## Abstract

Silicon (Si) waste generation is a critical issue in the development of semiconductor industries, and significant amounts of Si waste are disposed via landfilling. Herein, we propose an effective and high value-added recycling method for generating nitride nanoparticles from Si waste, such as poor-grade Si wafers, broken wafers, and Si scrap with impurities. Si waste was crushed and used as precursors, and an Ar-N_2_ thermal plasma jet was applied at 13 kW (300 A) under atmospheric pressure conditions. A cone-type reactor was employed to optimize heat transfer, and Si waste was injected into the high-temperature region between the cathode and anode to react with free/split nitrogen species. Spherical Si_3_N_4_ nanoparticles were successfully synthesized using isolated nitrogen plasma in the absence of ammonia gas. The crystalline structure comprised mixed *α*- and *β*-Si_3_N_4_ phases with the particle size <30 nm. Furthermore, the influence of ammonia gas on nitridation was investigated. Our findings indicated that Si_3_N_4_ nanoparticles were successfully synthesized in the absence of ammonia gas, and their crystallinity could be altered based on the reactor geometry. Therefore, the as-proposed thermal plasma technique can be used to successfully synthesize high value-added nanopowder from industrial waste.

## 1. Introduction

Si is the second most abundant element and the first most abundant solid material in the Earth’s crust and is the most widely used raw material in photovoltaic and semiconductor industries. However, the manufacturing process (particularly the ingot-cutting step) of crystalline Si wafers for application in solar cells and semiconductors can lead to significant Si waste generation in various forms, such as poor-grade or broken wafers and Si scrap with impurities. The amount of Si waste generated accounts for ~40% of the overall Si-based materials, exceeding a production rate of 10,000 tons/year. Approximately 101,750 tons of Si waste sludge was generated solely in 2010, with the annual amount rapidly escalating with the industrial development [1]. In the absence of commercial recycling technologies, Si waste disposal is mainly conducted by landfilling.

The development of effective recycling technologies for Si waste is essential because of the continuous strengthening of global environmental regulations. Contemporary methods focus on separating and recovering high-purity Si from industrial sludge that possesses a minimum Si nanoparticle content of 30% with a purity exceeding 9 N [2,3,4,5,6]. Our previous publication was focused on Si wafer waste treatment and Si nanoparticle production via radio frequency thermal plasma [7]. Recently, research interest has been focused on upcycling methods for producing high value-added materials from Si waste [8].

Si_3_N_4_ has been commercially produced since the 1850s, principally for application in high-temperature components, such as thermocouple tubes, rocket nozzles, boats crucibles for melting metals, and ceramic gas turbines [9]. As a high-temperature structure material, it exhibits numerous conducive properties, such as a high melting point (1900 °C), thermal shock resistance (i.e., low coefficient of thermal expansion; 1.4–3.7 × 10^−6^/K), strength (compressive strength; 524–5500 MPa, tensile strength: 60–525 MPa), and chemical stability. Si_3_N_4_ has been employed in diverse applications, such as for manufacturing engine parts in the automobile industry [10], bearing materials harder than metal, high-temperature materials (such as thrusters), and orthopedic devices [11,12]. Additionally, it is used as an alternative to polyether ether ketone and titanium owing to its hydrophilic and microtextured surface [13] and for fabricating abrasive and cutting tools because of their hardness [10], thermal stability, and resistance to wear. Furthermore, Si_3_N_4_ is used as an insulator, chemical barrier, and passivation layer in integrated circuits manufacturing.

Despite these advantageous properties, the commercial application of Si_3_N_4_ is hindered by factors such as its strong covalent structure, which requires high sintering temperatures, thereby increasing processing expenses. A practical strategy to overcome the limitations of high sintering temperatures is the reduction of the particle size, thereby increasing the surface area and energy, which enhance the sinterability. The use of Si waste to synthesize Si_3_N_4_ nanoparticles is a cost-effective process and has substantial significance in practical applications.

Herein, Si_3_N_4_ (*α*- and *β*-Si_3_N_4_) nanoparticles were synthesized by applying a thermal plasma jet to Si wafer waste [14,15,16,17,18]. Conventionally, the two primary methods used for Si waste processing are chemical dissolution and pulverization by applying physical force to produce nanopowders. However, these methods result in secondary environmental pollution or require the use of harmful gases (e.g., silane and boron chloride). Production of nanopowder, through the thermal plasma process, overcomes these complications [19]. Crushed Si wafer waste was effectively evaporated by applying a thermal plasma jet with a temperature exceeding 10,000 K, and the steep temperature gradient of the jet enabled rapid condensation at the nanoscale [20,21]. Additionally, Si_3_N_4_ nanoparticles were synthesized solely using nitrogen plasma gas without employing additional nitridation sources (e.g., ammonia gas (NH_3_)). The influences of reactors’ dimension and additional ammonia injection were investigated on the heat transfer for the formation of Si_3_N_4_ chemical bonding and crystal structure.

## 2. Materials and Methods

**Figure 1:**
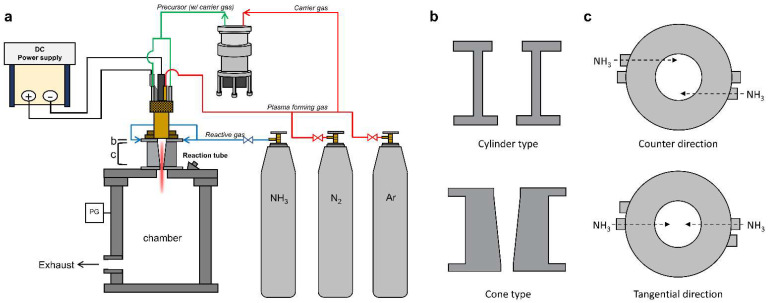
Illustration of experimental apparatus; (**a**) schematic diagram of the thermal plasma system, (**b**) geometry of the reaction tube (cylinder- and cone-type tubes), and (**c**) dimensions of injection nozzle for ammonia gas (counter and tangential directions).

A schematic of the thermal plasma jet system is illustrated in Figure 1a. It consists of a direct current (DC) power supply, a plasma-generating torch, a chamber with a reaction tube, and a powder feeder to inject the precursors. Thermal plasma was generated using a mixture of argon and nitrogen gas, and ammonia gas was additionally used as the reactive gas for nitridation. Micro-Si wafer waste powder (<38 μm) was injected into the thermal plasma jet torch via a nitrogen carrier gas using a splash-type powder feeder. The powder feeder accumulated scattered powder from the propeller and transported it to the outside, where it was released. Prior to thermal plasma expansion to the exterior of the torch, the interior was injected with Si powder through the two nozzles of the anode located within the same area. A detailed illustration was provided in a previous paper [22]. In this study, Si waste generated from an actual Si wafer production site was used as the precursor. Poor-grade Si wafers were crushed and sieved to produce particles with sizes smaller than 38 μm [7]. A reaction tube was used to enhance thermal transfer and chemical reactions between the plasma and precursors prior to the rapid thermal expansion within the chamber. The detailed structure of the tubes is shown in Figure 1b. The reaction tubes are cylindrical and conical in shape. Ammonia gas is injected at the top of the reaction tube in the counter or tangential direction, as shown in Figure 1c. The injection nozzles are located very closed to the exit of the plasma torch.

The experimental conditions are presented in Table 1. Thermal plasma, generated using argon, was flowed at a rate of 13 L/min. The flow rate of nitrogen fluctuated between 1.5 or 2 L/min, and the input power remained approximately constant between 13 and 14 kW. All experiments were performed at atmospheric pressure, and the flow was exhausted to the outside of the chamber. A cylinder-type reaction tube was used in Run 1, and a cone-type tube was utilized in the subsequent experiments (Runs 2–6). The feeding rate of the precursor was controlled from 0.1 to 0.7 g/min, and a constant rate of 2 L/min was maintained for the N_2_ carrier gas. The synthesis was performed exclusively using Ar-N_2_ thermal plasma during Runs 1–4. To investigate the influence of the highly reactive gas, ammonia gas was introduced at an inflow rate of 3 L/min in Runs 5 and 6, and the feeding direction was controlled by counter and tangential flow (Figure 1c). The synthesized nanoparticles were collected from the inner walls of both reactors. Most of the synthesized nanoparticles were condensed at the inner wall of the reactor, and only very few products were condensed on the inner surface of the chamber lid. The unreacted heavy precursor fell to the bottom of the chamber due to gravity.

The synthesized nanopowder was collected in a reaction tube. The crystalline structure was analyzed using X-ray diffraction (XRD, D/MAX 2200, Rigaku Co., Tokyo, Japan) with CuKα radiation (1.5406 Å) at 40 kV and 40 mA. The morphological characteristics and elemental composition at the microscale were examined using field-emission scanning electron microscopy (FE-SEM, S-4300, Hitachi Co., Tokyo, Japan) at an accelerating voltage of 15 kV. The nanostructures of the synthesized powders were analyzed via field-emission transmission electron microscopy (FE-TEM, JEM-2100F, JEOL Co., Tokyo, Japan) at an accelerating voltage of 200 kV using a carbon-coated copper holey grid.

## 3. Results and Discussion

**Figure 2:**
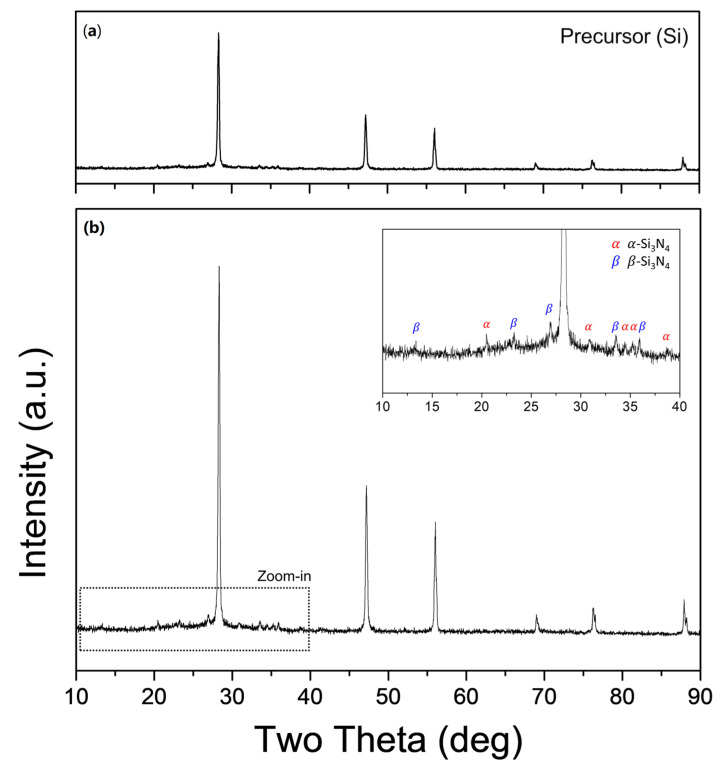
X-ray diffraction (XRD) patterns of (**a**) micro-Si powder as a precursor and (**b**) nanopowder synthesized in Run 1 (cylinder-type reaction tube). Inset shows an enlarged view of the graph (2θ = 10–40°).

Figure 2 shows the XRD patterns of the precursor (micro-Si wafer waste powder) and the powder synthesized in Run 1 (cylinder-type). Sharp peaks corresponding to crystalline hexagonal Si are observed, and an enlarged view of the patterns provided in the inset shows blunt peaks for *α-* and *β-*Si_3_N_4_. The main peaks of *α-* and *β-*Si_3_N_4_ are detected at 20.46° and 27°, respectively. The nitrogen gas in the thermal plasma jet reacted with the vaporized Si precursor material. The nitrogen particles were sequentially dissociated by the strong electric field and then re-associated via rapid temperature reduction owing to the thermal expansion at the exit of the torch. Sufficient time was available for obtaining Si vapor prior to molecular re-association because the Si precursor was injected into the interior of the torch.

**Figure 3:**
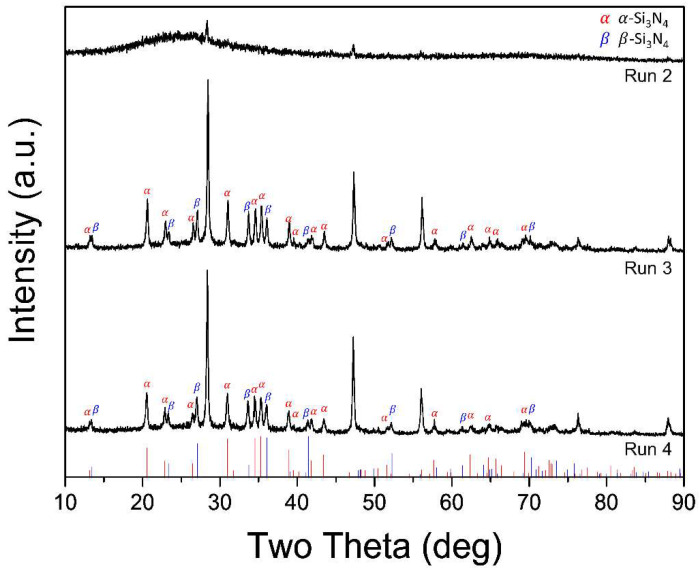
XRD patterns of nanopowders synthesized in Runs 2, 3, and 4 (cone-type reaction tube).

To enhance the heat transfer between the plasma and the precursors, we designed a cone-type reaction tube. The cylinder-type reaction tube has narrow entrance and exit jaws (Figure 1b), as demonstrated by the smaller diameter of the entrance and exit compared to the midpoint diameter. This aids the formation of a potent vortex near the reactor wall, whereas the plasma expands thermally from the torch nozzle. Fluid stagnation due to eddies occurs owing to the abrupt narrowing of the outlet. The cone-type tube is designed to achieve a gradual decrease in the tube diameter without using any jaws (Figure 1b), to prevent fluid stagnation and the rapid quenching of the plasma jet. The feeding rate of Si powder was maintained at 0.1, 0.4, and 0.7 g/min in Runs 2–4, respectively, to increase the density of Si vapors. Figure 3 shows the XRD patterns of nanopowders synthesized in Runs 2–4. Reference peaks are located at the bottom of the graph (red vertical line: *α-*Si_3_N_4_; blue vertical line: *β-*Si_3_N_4_). Under the 0.1 g/min Si feeding condition (Run 2), an amorphous shoulder is observed with weak crystalline Si peaks. Peaks of *α-* and *β-*Si_3_N_4_ are observed for the nanopowders synthesized in Runs 2 and 3, respectively. The vapor density of the precursor material increases proportionally with the feeding rate. This allows early supersaturation to occur, and nuclei form and crystallize over longer time intervals. However, because an increase in the feeding rate also lowers the heat or energy density of the plasma for the vaporization of the precursor, the feeding rate of the precursor should be appropriately adjusted.

The crystallites sizes were calculated by the Scherrer equation:

$$D=\frac{K\lambda }{\beta cos\theta }$$

where *D* is the crystallite size of the synthesized *α-* and *β-*Si_3_N_4_; *K* is the Scherrer shape factor (*K* = 0.9); *λ* is the X-ray wavelength (*λ* = 1.5406 Å); *β* is the full width at half maximum (FWHM) of the main peak; *θ* is the Bragg angle of the main peak. *α-*Si_3_N_4_ was detected at 2*θ* values of 20.64° and 20.98° for Runs 3 and 4, respectively, and the crystallite sizes were determined as 26.82 and 13.30 nm, respectively. *β-*Si_3_N_4_ was calculated at 2*θ* values of 27.14° and 26.9° for Runs 3 and 4, respectively, and the crystallite sizes were estimated as 21.23 and 19.06 nm, respectively. These results confirm the nanocrystalline characteristics of the synthesized Si_3_N_4_ nanoparticles.

**Figure 4:**
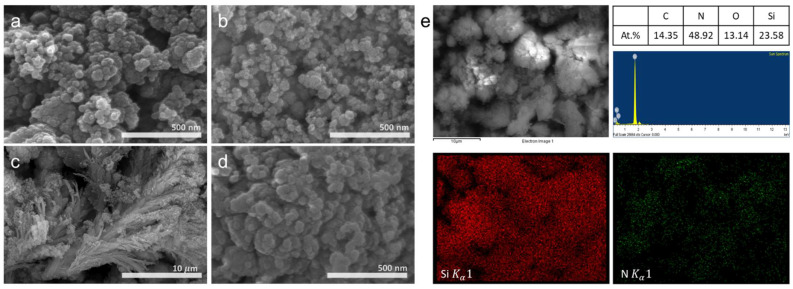
(**a**–**d**) Field emission (FE) Scanning electron microscopy (SEM) images of nanopowders synthesized in Runs 1–4, and (**e**) elemental mapping and composition of the nanopowder obtained in Run 3.

The morphological characteristics were analyzed via FE-SEM as shown in Figure 4a–d (Runs 1–4). The synthesized nanopowder is composed of spherical nanoparticles with sizes smaller than 100 nm. Feather-like materials are observed on spherical nanoparticles obtained in Run 3. Elemental mapping was performed to examine the chemical composition of the nanopowders obtained in Run 3. Figure 4e shows that nitrogen and Si are uniformly distributed in all particles. The bulk composition ratio of Si to nitrogen (N/Si) is 2.07.

Furthermore, the commercial and synthesized Si_3_N_4_ samples were compared to analyze the broad sample area. The elemental composition of the synthesized nanoparticles is listed in Table 2, wherein a comparison with commercial *β-*Si_3_N_4_ micropowder is presented. The N/Si ratio of commercial powder is 2.11, and the corresponding ratios of the synthesized nanoparticles are between 1.45 and 1.69. The N/Si ratios are comparable for all nanoparticles, and a marginally lower nitrogen content is observed for as-synthesized samples than commercial Si_3_N_4_ owing to the Si residue. Additionally, in Run 2, amorphous Si_3_N_4_ is considered to have formed, as an amorphous shoulder is observed in the XRD pattern of the corresponding nanopowder.

**Figure 5:**
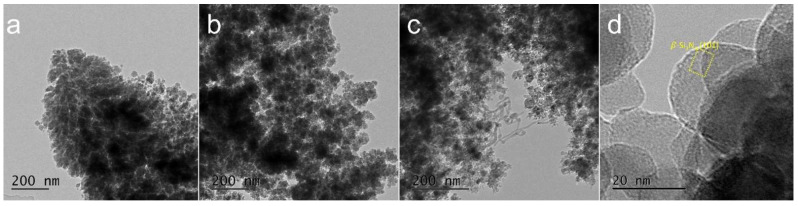
FE-transmission electron microscopy (TEM) images of nanopowder synthesized in (**a**) Run 2, (**b**) Run 3, and (**c**,**d**) Run 4.

Figure 5 shows FE-TEM images of the synthesized nanoparticles depending on the feeding rate of Si waste. As shown in FE-SEM analysis, spherical nanoparticles with sizes less than 30 nm are observed in all experiments. Several nanowires observed after Run 4 are estimated to be Si nanowires based on previous experiments [23]. Choi et al. reported the synthesis of Si nanowires using a strain of molten Si with a relatively low thermal conductivity compared with that of light metals, using the same thermal plasma system employed in this study. Additionally, lattice fringes of the *β-*Si_3_N_4_ nanostructure were analyzed, and the results are presented in Figure 5d, which exhibits numerous amorphous spherical nanoparticles.

**Figure 6:**
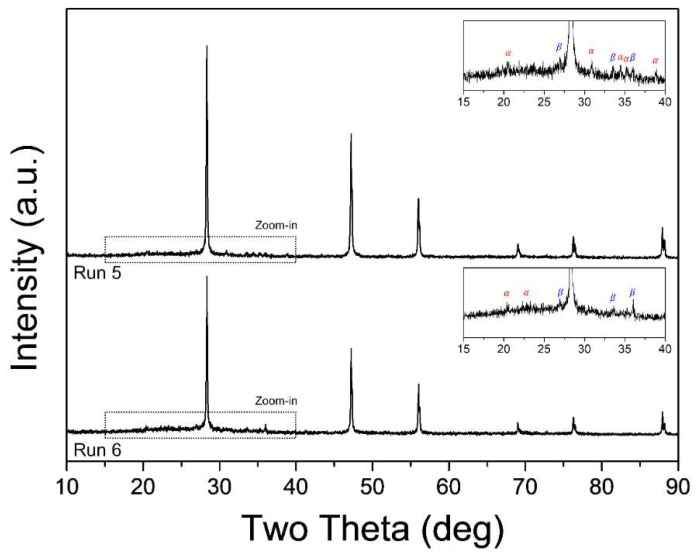
XRD patterns of nanopowders synthesized in Runs 5 and 6 (cone-type reaction tube with NH_3_ gas addition). Inset presents an enlarged view of the graph (2θ = 10–40°).

**Figure 7:**
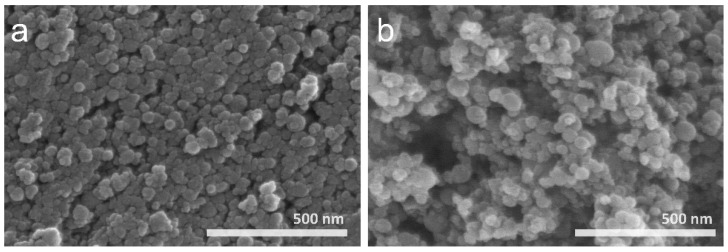
FE-SEM images of nanopowder synthesized in (**a**) Run 5 and (**b**) Run 6.

Ammonia gas is conventionally used as a reactive source for nitridation in thermal plasma processing, despite its toxicity. The diatomic nitrogen molecule dissociates at temperatures higher than 7000 K, and the decomposition temperature of NH_3_ molecules is approximately 600 K [24]. Therefore, nitridation using ammonia gas is preferable to synthesis by nitrogen. Occasional nitridation is caused by nitrogen in the ammonia molecules, whereas hydrogen contribute to crystal growth. Therefore, ammonia gas was injected to investigate its effects on the synthesis and crystal growth of Si_3_N_4_ nanoparticles in Runs 5 and 6. The feeding rate of Si waste was maintained at 0.1 g/min. The XRD patterns of the synthesized nanopowder are shown in Figure 6. Crystalline peaks of Si, which are presumed to originate from the unvaporized residue, are observed, and blunt peaks of *α-* and *β-*Si_3_N_4_ are noted in the enlarged image of the XRD pattern for both Runs 5 and 6. No notable difference is observed between the counter and tangential injections of NH_3_ gas. In comparison with the XRD pattern for Run 2 (similar experimental conditions without ammonia injection), the vaporization of the Si precursor is deteriorated by cold ammonia injection. Figure 7 indicates that the morphology and size of the synthesized nanoparticles are similar to those of the nanoparticles obtained in Runs 2–4. Ammonia gas was injected to provide additional reactive components for nitridation and enhance crystallinity; however, the injection of additional cold gas quenched the plasma jet and consumed energy owing to the decomposition of ammonia molecules [24]. The ammonia gas was injected perpendicular to the direction of plasma jet emission (axial direction), which was able to reduce the plasma jet volume. Since the ammonia gas flow rate of 3 L/min has a 20 vol% compared to the plasma forming gas flow rate of 15 L/min, the increase of total fluid flow rate leads to the velocity increase and complex fluid flow inside of the reactor. The quenching effect must have been quite large. In other words, the relatively high-temperature area decreases with the injection of additional cold gas that does not affect plasma generation. It was estimated that the ammonia gas creates a suppressive environment for the evaporation of precursor and the synthesis of Si_3_N_4_ due to the reduction of residence time at the high temperature region.

**Figure 8:**
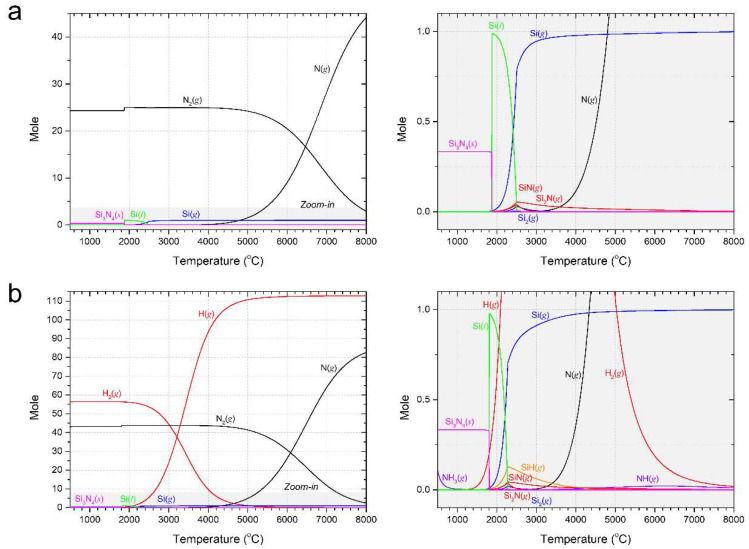
Chemical composition at the thermodynamic equilibrium (**a**) Si-N_2_ system and (**b**) Si-N_2_-NH_3_ system. An enlarged view of the graph (y-axis: −0.1–1.1) is presented on the right side of each figure.

**Figure 9:**
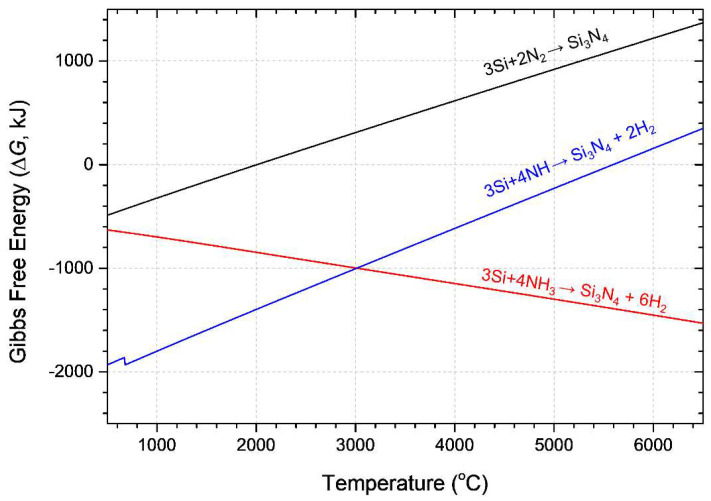
Change in Gibbs free energy (Δ*G*) of the nitridation of Si by nitrogen and ammonia species.

The influence of ammonia gas was thermodynamically analyzed using FactSage software (ver.6.4, Thermfact/CRCT&GTT-Technologies, Montreal, QC, Canada/Frankfurt, Germany). Stable chemical molecules of the Si–N_2_ system (Figure 8a) and Si–N_2_–NH_3_ systems (Figure 8b) were observed at the thermodynamic equilibrium state, as shown in Figure 8. The molecular ratios of Si, N_2_, and NH_3_ were controlled at flow rates of 0.1 g/min and 2 and 3 L/min, respectively, and were maintained at 1:25:37.6. Because the gas phase in the precursor is highly occupied, most of the stable species are hydrogen and nitrogen (N_2_, N, H_2_, and H). In the Si-N_2_ system (enlarged image), SiN and Si_2_N generation commences at temperatures above 2000 K, whereas in the Si-N_2_-NH_3_ system (enlarged image), NH_3_ and decomposed reactive gas NH coexist with SiN and Si_2_N gases. Additionally, a small number of nitrogen-containing molecules (such as NH_2_, N_2_H_2_, and N_2_H_4_) is formed. The calculated reaction spontaneity is shown in Figure 9. The synthesis of Si using nitrogen and ammonia molecules (NH_3_ and decomposed NH) was collated as a function of temperature. Nitridation with ammonia molecules is superior to that with nitrogen for the entire temperature range. Furthermore, hydrogen enhances thermal transfer and further crystallization owing to its higher thermal conductivity than that of argon and nitrogen plasma forming gas.

The target material, nitride, seldom reacts with N_2_ gas because the change in Gibbs free energy (∆*G*) has positive values at temperatures below its dissociation temperature. However, nitridation is spontaneous, with dissociated nitrogen radicals (N) in the same temperature range. The reaction is hampered by the recombination of N radicals into N_2_ (N + N → N_2_). ∆*G* for recombination is located slightly below the ∆*G* for nitridation. However, ∆*G* for nitridation by N ions (N^+^) is lower than that for the recombination of N ions with an electron. Herein, nitrogen gas was used as the plasma-forming gas. Therefore, the injected N_2_ molecules were exposed to a strong electrical field and formed N ions via ionization [25] (N + e → N^+^ + 2e with a threshold energy of 14.5 eV, and N_2_ + e = N^+^ + N + 2e with a threshold energy of 24.3 eV). In a DC thermal plasma torch, although nitrogen ionized at high temperatures undergoes a recombination reaction to form N atoms below 10,000 K, it can exist at jets with temperatures lower than 7000 K [26,27,28]. Assuming that the temperature field at the torch exit has a maximum of 13,000 K and a minimum of 1000 K based on a previous report [24], the area around the torch exit, where the Si precursor is injected, contains a sufficient number of N ions owing to the continuous injection of N_2_ gas. The torch configuration also contributes to the reaction between the Si precursor and ionized N ions because it allows the Si to penetrate the core of the plasma flame because of the injection before the thermal expansion of the plasma at the torch nozzle, which contains the highest concentration of ionized N ions in the system. Consequently, nitridation with N ions and electrons results in the lowest negative Δ*G* values among the various nitridation reaction paths.

Notable results of this study can be listed as follows. First, Si_3_N_4_ nanoparticles were synthesized in the absence of toxic ammonia gas and solely using nitrogen plasma forming gas, and second, the efficiency of the reactor design enhanced the crystallization of Si_3_N_4_.

The industrial application of Si_3_N_4_ nanoparticles requires the existence of *α-*Si_3_N_4_. In previous studies, although toxic ammonia gas was used for nitridation, *α*-phase formation was strongly dependent on the NH_3_/precursor ratio [14,15,16,17]. This ratio possibly affects the temperature distribution of the plasma flame and provides an etching effect through hydrogen production via the decomposition of ammonia. In future studies, we would like to investigate the improvement of the *α-*Si_3_N_4_ phase in the absence of ammonia gas.

## 4. Conclusions

Si_3_N_4_ nanoparticles (with *α-* and *β-*Si_3_N_4_ phases) were effectively synthesized using Si wafer waste employing a DC plasma jet system. The nitridation source was nitrogen, which originated from the plasma forming gas. Poor-grade and broken wafers and Si scrap with impurities were pulverized to form particles with sizes smaller than 38 μm, and the powder was injected into the plasma region within the torch. Si_3_N_4_ nanoparticles with low crystallinity were synthesized in a cylinder-type reaction tube. Subsequently, the crystallinity was significantly enhanced using the cone-type reaction tube owing to the prevention of rapid condensation via thermal expansion and the enhancement of heat transfer between the plasma and Si precursors. Amorphous Si_3_N_4_ nanoparticles were obtained at a significantly low Si feeding rate (0.1 g/min) owing to the short growth time caused by late supersaturation. The crystallinity increased proportionally with the feeding rate (0.4 and 0.7 g/min). The crystal structure of the synthesized Si_3_N_4_ nanoparticles comprised mixed *α* and *β* phases, and the crystallinity of the *α* and *β* phases was almost the same considering that they had similar crystallite sizes. The as-synthesized Si_3_N_4_ comprised spherical particles with sizes less than 30 nm. In comparison to the experiment with ammonia gas, nitridation efficiently occurred using nitrogen molecules originated from the plasma-forming gas without using toxic reactive species in the thermal plasma jet system. Furthermore, nitridation and crystallization were improved by altering the reactor design to enhance heat transfer. Consequently, the as-proposed thermal plasma technique can be used to effectively synthesize high value-added nanopowders while processing industrial waste. However, the vaporization of Si waste should be improved, and accordingly, the purity of Si_3_N_4_ should be enhanced. We expect that these aspects can be realized using multiple plasma reactors in the industry. Currently, the techniques to improve the lifetime for the stability and durability of DC thermal plasma torch are sufficiently advanced; therefore, the stable operation of the device to obtain a continuous discharge for ~1000 h is feasible.

## Figures and Tables

**Table 1:** Experimental conditions for synthesizing Si_3_N_4_ nanoparticles using thermal plasma.

Parameters	Run 1	Run 2	Run 3	Run 4	Run 5	Run 6
Reactor type	Cylinder-type	Cone-type				
Plasma forming gas	13 L/min Ar 1.5 L/min N_2_				13 L/min Ar 2 L/min N_2_	
Feeding rate of Si	0.5 g/min	0.1 g/min	0.4 g/min	0.7 g/min	0.4 g/min	
Feeding rate of NH_3_	None.				3 L/min	
Feeding direction of NH_3_	None.				Counter	Tangential

**Notes.** Input power was almost constant between 13–14 kW at fixed 300 A in all Runs. Chamber pressure was fixed at atmospheric pressure. The carrier gas for the injection of silicon precursor was used as nitrogen gas with 2 L/min.

**Table 2:** Elemental composition of commercial and synthesized Si_3_N_4_ powders in Runs 2, 3, and 4.

(Unit: at. %)	Commercial *β-*Si_3_N_4_	Run 2	Run 3	Run 4
Si	27.68	23.59	25.7	20.03
N	58.45	36.96	37.4	34.03
N/Si ratio	2.11	1.56	1.45	1.69

## Data Availability

Data supporting reported results is available and provided at reasonable request.
